# Crown- and Bridge-Work

**Published:** 1894-05

**Authors:** Cecil P. Wilson


### CROWN- AND BRIDGE-WORK.[1]

BY CECIL P. WILSON, D.M.D.

I do not know that I have anything especially new to offer on this subject. It seems to me, however, that sufficient time has elapsed since the introduction of artificial crown- and bridge-work to enable the profession to come to some conclusions regarding its merits and defects.

The first piece of bridge-work I ever saw was in a dental office where this branch of the profession was carried on on an extensive scale. It was in the mouth of a paid attendant,—a sort of walking show-case,—who kept his mouth as clean as possible, and held himself in readiness to exhibit it to those who desired ocular demonstration of the new methods of inserting artificial dentures. This piece of bridge-work was in the upper jaw, and consisted of fourteen teeth, having for anchorages the cuspid and molar teeth. This piece, as I remember it, did not appear to be a success from an æsthetic point of view. The absence of artificial gum failed to give a proper contour to the mouth, and the teeth were set so closely together, and with such extreme regularity, it had an artificial look. In a mechanical sense, however, it was well put together and made a handsome showing, was novel and interesting to look at, and seemed to suggest great possibilities for the future.

The criticisms of permanent bridge-work show a wide diversity of opinion. One man says, "But as a practical fact, permanent bridges are simply the nastiest things ever made to do duty as substitutes for nature's work;" while another says, "The truth is, a skilfully constructed and properly adapted bridge is one of the most elegant and cleanly operations in dentistry, and is the most perfect substitute at present known for supplying the loss of the natural teeth. In the *Dental Cosmos* of January, 1884, Dr. J. L. Williams of Boston, one of the past presidents of this society, gave his opinion as follows:

"*To the Editor of the Dental Cosmos:*

"Dear Sir,—To correct any misapprehension, I wish to say in regard to the so-called bridge-work, now somewhat discussed, that from observations of a variety of fixtures of that sort since 1844 to the present time (some of them very skilfully made), I do not recom-

[1] Read before the Academy of Dental Science, January 3, 1894.

mend as cleanly and wholesome in the mouth any extensive appliance that can not be removed daily for cleaning, and I consider as unpardonable the practice of cutting of good and useful teeth merely to use their roots for supports of a 'bridge.'"

I presume our Boston Dr. Williams did not wish to be responsible for the sayings of the New Haven Dr. Williams, both having the same initials, and he took occasion to define his position in the matter. At all events, the opinion of a man who has been a close observer in our profession for forty years and more is worth something.

Dr. Williams's reference to the cutting of good and useful teeth reminds me of a dentist who lost a central incisior in early life and wore a porcelain substitute, mounted on a suction plate, for a great many years. When bridge-work was introduced the plate was discarded, the good central cut off, and the pulp destroyed. Subsequently, a Richmond crown (with dummy central attached) was constructed, which, I believe, has been worn for about ten or twelve years, and with which he expresses himself as completely satisfied.

This gentleman is an intelligent observer in good standing in our profession, and I often wonder if he is willing to take the responsibility of practising such methods on his patients. There are cases where such heroic treatment might not be considered unobjectionable, but, as a rule, the cutting off of sound teeth to make way for bridge-work is reprehensible practice and should be strongly condemned. I do not make extensive pieces of bridge-work, for reasons which I shall give later, though my experience leads me to believe that small pieces can be used to good advantage. For a time I was quite enthusiastic over permanent bridge-work, but I discovered certain objections, three of which are, difficulty of cleaning, risk of stopping up pulpless teeth, which are liable to give trouble, and liability of porcelain crowns to fracture. I know there are enthusiastic advocates of permanent bridge-work, who think such fixtures are as easily cleaned as the natural teeth, but my observations have led me to form a different opinion. A few weeks ago I took a large piece of calculus from the mouth of a fastidious person, who nearly fainted at the idea of having such a thing in her mouth, and I am persuaded that there are many such people now wearing pieces of bridge-work, unsuitable from a hygienic point of view, either because of unfavorable conditions or faulty construction.

If such individuals could examine their mouths with the critical eye of a dentist, I think they would discard such fixtures and ask for a substitution of some other kind. Notwithstanding our advanced knowledge of the care of pulpless teeth, and the numberless therapeutic agents at our command, it must be admitted that there are pulpless teeth which cannot be permanently filled, and a good many cases where a consideration of diathesis and constitution would seem to warrant a conservative course.

All such cases are unsuited to permanent bridge-work. There are veterans of the old school who still carve their own teeth, and who look with disdain, and not without reason, upon the teeth furnished us by the manufacturers of the present day. Silex is the mainstay and backbone of tooth-body. Moulded teeth are generally lacking in this essential ingredient, hence they are weaker than they ought to be and liable to fracture. Occasionally I come across an unusually weak piece of porcelain. Some years ago I made an upper set of plain teeth on rubber for a gentleman, who came back in a few days with one of the teeth broken. He assured me the teeth had not been subjected to any undue strain, and that the tooth had broken off while eating his dinner, which consisted of soft food. Inside of two weeks two other teeth broke off in the same way. I then took off all the teeth and replaced them with a new set, which was worn, without any further accident, until his death some seven or eight years after. If this had been a case of permanent bridge-work, it would have been exceedingly trying to both patient and dentist. To my mind, the liability of porcelain teeth to fracture is a serious objection to the use of permanent bridge-work. For single crowns I use the Richmond method, the arguments in favor of this system of crowning teeth, to my mind, far outweighing any that can be brought against it. By the use of this crown a larger proportion of tooth-substance can be saved than by any other. With all porcelain crowns, on the contrary, it is necessary to cut away the tooth to the level of the gum. In making Richmond crowns, I use eighteen-karat gold, from thirty-two to thirty-four gauge. I do not care for the stretching or elastic properties found in the softer kinds of gold, and while it is a little more difficult to adapt the stiffer gold to the root, it has more stability, and, being thinner, offers less chance for accumulations around it. The arguments against it are the showing of the gold band and difficulty of construction. I am occasionally asked by a patient if the band will show, but seldom have any complaints when the crown is completed. The length of time consumed in the construction of a Richmond crown depends entirely upon the operator. A man who bends a band or ferrule to a root for the first time is apt to find his calculations wide of the mark; but, with constant practice, his eye becomes so trained that the size of the root can be taken in almost at once, and the first fitting is apt to be pretty nearly correct. Those who try to make Richmond crowns from models must inevitably fail. In the mouth we have the yielding gum, and the natural root which offers resistance to the gold without any danger of chipping away, whereas, in casts of plaster and fusible metal, these conditions are entirely wanting; therefore, the bands or ferrules should in all cases be fitted to the natural root and the two ends joined together with solder before the patient leaves the chair. In order to convey to your minds as clearly as possible what I have to say, I have made a few drawings of pieces of bridge-work. It will be observed, in the first place, that the central incisor is missing, and between the other roots and teeth there are wide spaces. The bridge consisted of three teeth extending from the left central to the right lateral. I was somewhat at a loss to know how to join these teeth together. The ordinary method of constructing bridge-work at the time this was made consisted of placing the teeth side by side and joining them by their "backings." This method of procedure was impossible in this case, owing to the wide spaces between the teeth.

The difficulty was overcome, however, by fitting a stout piece of platinum and iridium wire to the palatal surfaces of the teeth, and bearing on the gum. The wire was closely adapted to the gum, and, from the front of the mouth, clear spaces could be seen, and the form of attachment was entirely out of sight. Care was taken to trim the circumference of each root, in order that the bands might fit as accurately as possible. A Richmond crown was made for the left lateral incisor, which was to be placed in position independent of the bridge. Crowns were then made for the left central and right lateral, placed in position with a dummy tooth in between, then removed and finished in the usual manner. The bands were perfectly adapted to the roots, and fitted with such accuracy that some force was necessary to remove the crowns.

On attempting to try in the completed bridge, I found it would not go in place without a good deal of coaxing and pushing, and when finally placed in position the bands were slightly bent from their original shape. This opened my eyes to an unavoidable imperfection in permanent bridge-work. The person never was born whose teeth are on exactly parallel lines with each other; consequently no piece of bridge-work can be constructed which can accurately, in the true sense of the word, fit the roots upon which it is placed. If a single crown is made with a poorly-fitting band, the result is more or less inflammation about the gum, exposure of the band, and possible irritation of the peridental membrane.

Now, this condition of things must inevitably be present to a greater or less extent in permanent bridge-work. As I have said before, the bands fitted accurately; if, however, after forcing the completed bridge into place, I had cut the platinum wire which held the crowns together, and separated them, I should probably have found them somewhat loose, with an adaptation of the bands to the roots, less perfect than in the case of the lateral incisor, which was made independent of the bridge. This bridge was worn about two years, when it became loose. Before replacing it, holes were bored through the gold in the base of each crown to allow for overflow of cement, the holes afterwards being stopped up with gutta-percha. This was worn about two years, when the cement gave out a second time. The third time red gutta-percha, such as is used for base-plates, was substituted for oxyphosphate of zinc, which had been previously used, and for a time everything went on well. It will be observed, in this case, there is considerable over-bite, and the lower teeth acting as levers forced the bridge out of place, necessitating a removal of the bridge a third time. I then cut the wire which connected the right lateral with the dummy central, smoothed down the projecting points, and replaced the teeth with cement.

I saw the case some three months since, and at that time it seemed to be doing well. The left lateral incisor crown remains in just the same condition as when first placed in position. This crown was a success, for the reason that, being unhampered by any other tooth, it was possible to push it directly into place. There was no straining or bending of the band which could cause any deviation from its original form, thus assuring a continuity of surface and an hermetically sealed root, which prevented the secretions from coming in contact with the cement.

Another, I constructed, consisted of a piece extending from the right central to the right molar; as can be seen by the sketch, the right central and second bicuspids are missing. Crowns were made for the cuspid and first bicuspid roots, also dummy crowns to take the place of the missing central and second bicuspid. To this latter was also soldered a platinum pin which projected into a cavity in the first molar tooth. This cavity was afterwards filled around the pin and assisted in maintaining the stability of the piece. The central was connected with the cuspid by a gold bar, curving around the lateral incisor and bearing on the gum. When finished, this made a complete bridge from the right central to the first molar tooth. It was impossible to thoroughly clean the bar around the lateral, and, as a consequence, disintegration of the tooth went slowly on until it finally broke off. Instead of taking off the entire bridge for the purpose of adding a new lateral, I cut the bridge in between the cuspid and bicuspid, made a new cuspid and lateral, to which was added the dummy central, making another bridge independent of the other.

When the cuspid crown was removed I found the root and cement in perfect condition, notwithstanding the fact that this bridge had been worn a year longer than the previous bridge to which I have alluded. Another piece of bridge-work, which has been worn about ten years, is very similar to the last, with the exception of the gold bar, which curved around the canine instead of the lateral, as previously mentioned. I saw this case about two years ago, and there were indications of disintegration around the cuspid tooth; otherwise the piece was apparently in good condition.

This last sketch is supposed to represent a case for which I made a piece of removable bridge-work. There are the four incisors, a first left bicuspid, second bicuspid root, and one molar tooth. With the exception of the bicuspid root, all the teeth are alive. Several of the molar teeth in this case were lost in early life, and, as a result, the upper jaw is somewhat contracted and there is a slight protrusion of the lower jaw. A few years ago the protruding lower teeth began to gradually wear away the labial aspect of the incisors, until they assumed an unsightly appearance, and an artificial piece of some kind was rendered imperative. Perhaps it may increase the interest in this case a little to say that I am speaking of my own mouth. The labial surfaces of the incisor teeth were worn away as represented in the sketch. It seemed wicked to destroy the pulps of these perfectly sound teeth, and, being averse to extraction, I decided to make a piece of bridge-work. A gold cap was made for the bicuspid tooth, and the inclined plane of the incisor teeth smoothed down as much as possible. Richmond crowns were then made of carved teeth for the lateral incisors, an all porcelain tooth to take the place of the missing cuspid, and two pieces of porcelain which were made to fit accurately the central incisors. The five teeth and bicuspid cap were then joined together in the usual way and finished. The palatal surfaces of the carved incisor teeth were only half as thick as on the labial surfaces, and this necessarily left the porcelain weak in just the place where the most strength was needed. After the piece had been worn as a removable bridge for about six months, I decided to grind away more of the natural incisors to admit of larger and stronger porcelain teeth and to make more sure of a larger and more extensive piece of bridge-work. Gold caps were then made for the molar and bicuspid teeth, porcelain teeth were made to fill all the vacant spaces, and the result was a bridge extending entirely around the mouth and articulating perfectly with the twelve teeth in the lower jaw. In thinking over what I wanted to say this evening, I realized that it was easy to drift into considerable detail which would be tedious and uninteresting to my hearers; therefore, I have not touched upon several points, properly belonging to this subject, which will probably be brought out during the discussion which follows.

